# Supplementary material for: Association between oral microbiome and seven types of cancers in East Asian population: a two-sample Mendelian randomization analysis
Source: Front Mol Biosci. 2023 Nov 21;10:1327893. doi: 10.3389/fmolb.2023.1327893 (PMC10702768; doi:10.3389/fmolb.2023.1327893)
Supplement: Supplementary file 1 [file DataSheet1.ZIP › Supplementary Materials/MR plots of saliva/saliva═╝/ovarian cancer/pheno.143_to_ovarian cancer_leave_one_out.pdf]

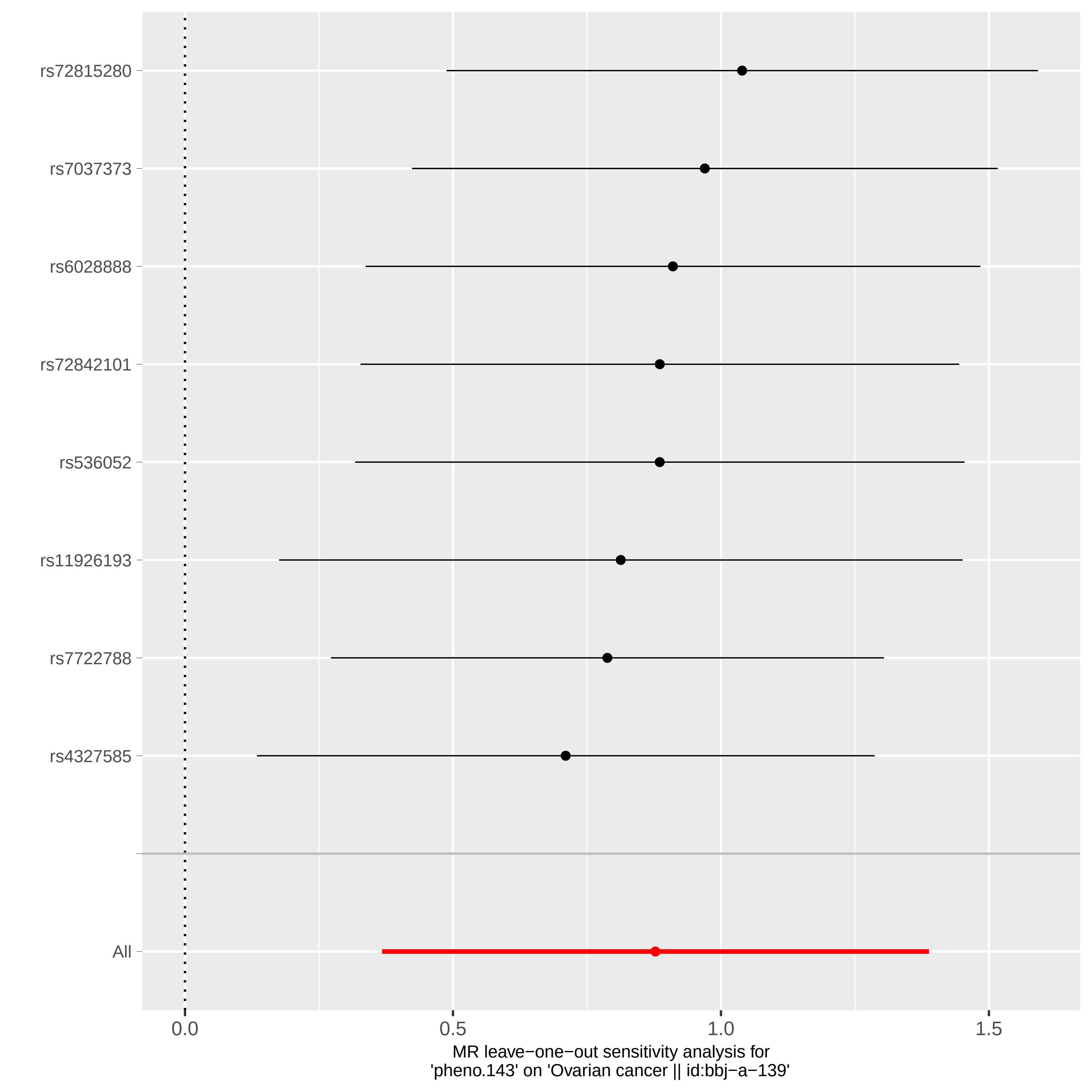
rs72815280
rs7037373
rs6028888
rs72842101
rs536052
rs11926193
rs7722788
rs4327585
All
0.0
0.5
1.0
1.5
MR leave−one−out sensitivity analysis for
'pheno.143' on 'Ovarian cancer || id:bbj−a−139'
